# Modelling of the material flow of Nd-Fe-B magnets under high temperature deformation via finite element simulation method

**DOI:** 10.1080/14686996.2017.1362589

**Published:** 2017-08-22

**Authors:** Yen-Ju Chen, Yen-I Lee, Wen-Cheng Chang, Po-Jen Hsiao, Jr-Shian You, Chun-Chieh Wang, Chia-Min Wei

**Affiliations:** ^a^ Metal Industries Research and Development Centre, Kaohsiung, Taiwan; ^b^ Department of Physics, National Chung Cheng University, Chia-Yi, Taiwan; ^c^ Department of Mold and Die Engineering, National Kaohsiung University of Applied Sciences, Kaohsiung, Taiwan

**Keywords:** Hot-deformed Nd-Fe-B magnet, finite element simulation, 60 New topics/Others, 400 Modeling/Simulations, 106 Metallic materials

## Abstract

Hot deformation of Nd-Fe-B magnets has been studied for more than three decades. With a good combination of forming processing parameters, the remanence and (BH)_max_ values of Nd-Fe-B magnets could be greatly increased due to the formation of anisotropic microstructures during hot deformation. In this work, a methodology is proposed for visualizing the material flow in hot-deformed Nd-Fe-B magnets via finite element simulation. Material flow in hot-deformed Nd-Fe-B magnets could be predicted by simulation, which fitted with experimental results. By utilizing this methodology, the correlation between strain distribution and magnetic properties enhancement could be better understood.

## Graphical abstract

**Figure:**
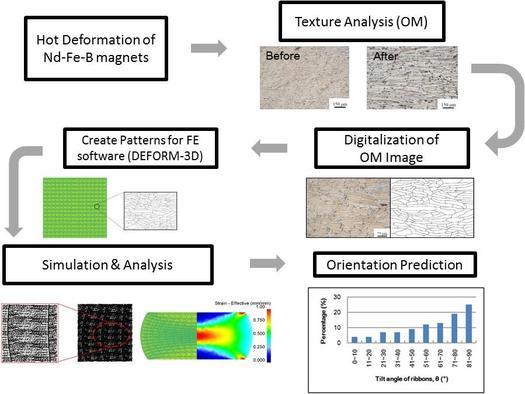

## 1. Introduction

Nd-Fe-B magnets were utilized mainly in motors for their outstanding magnetic properties. Besides bonded magnets and sintered magnets, the study of a third way to manufacture high-performance Nd-Fe-B magnets began in 1985[1]. Lee proposed that rapid-quenched Nd-Fe-B ribbons could be compacted to a fully dense magnet with a preferred magnetization direction (easy magnetization axis) parallel to the hot deformation (HD) direction. The HD magnet exhibited a remanence (Br) of 13.5kG, and an intrinsic coercivity (iHc) of 11kOe, which yields to a maximum energy product (BH)_max_ around 40MGOe. Over the years, research groups have tried to master the HD processing techniques, and to make HD magnets with better properties by different schemes. For example, Yoshida et al. [2] tested several HD processes (namely, cylinder upsetting, backward ring extrusion, forward ring extrusion, and forward bar extrusion), and obtained HD Nd-Fe-B magnets with different geometries and different magnetic properties. They suggested that magnetic properties (especially Br values) are dependent on the strain states of HD magnets. Furthermore, they found the upsetting process gave the best result because of the suitable strain states. The strain states in upsetting include three components: one compressive and two tensile.

Since then, Yoshida et al. [3] have tried to improve the magnetic properties of HD Nd-Fe-B magnets through compound design. They studied the deformation behavior of HD Nd-Fe-B magnets since strain states of material may greatly influence magnetic properties. They suggested that superior Br values could be achieved at an optimized strain rate for HD magnets. In their research, Arrhenius’ equation was chosen to describe the relations between flow stress and strain rates. Even now, research groups are still finding different schemes to optimize the magnetic properties through upsetting experiments. Table 1 shows research results related to HD Nd-Fe-B magnets (mainly on upsetting [11,13–17], but some of the works also focus on backward ring extrusion [9,10,12]). It can be seen that the Br values have been improved to 14.9kG [15], and the record of (BH)_max_ values had been increased to 53.7MGOe [17] in the central part of the magnet.

**Table 1.** Magnetic properties of HD Nd-Fe-B magnets made by different groups.

Research Group	Process Temperature (℃)	Deformation Ratio (%)	Remanence (kG)	Coercivity (kOe)	(BH)_max_ (MGOe)
Lee [1]	700	50	13.5	11.0	40.0
Yoshida [2]	700 ~ 750	55	12.8	14.9	39.3
Yoshida [3]	750	60	12.8	16.5	40.2
Kasai [9]	700	70	12.6	15.2	37.8
Grunberger [10]	750	64	13.0	15.0	N/A
Kim [11]	N/A	80	13.9	10.9	44.2
Kirchner [12]	750	67	13.0	17.0	41.0
Hioki [13]	780	75	13.3	12.7	39.0
Hou [14]	800	73	13.3	11.4	38.8
Zhang [15]	675	75	14.9	9.3	53.0
Lee [16]	780	70	13.8	15.7	46.5
Zhu [17]	N/A	70	14.7	9.6	53.7

The deformation ratios shown in Table 1 give the impression that with sufficient deformation in HD processes, Br and (BH)_max_ values of magnets could be much elevated. Hioki et al. [13] studied the effect of deformation ratios on magnetic properties. From their results, it can be inferred that the Br values are elevated to 85% of saturation remanence (Bs) value when the deformation ratio reaches 50%. When the deformation ratio exceeds 70%, the elevation in Br value is not so significant due to excessive grain growth under high temperature. Therefore, many researchers [9,11,13–17] prefer the deformation ratio around 70% in order to maximize the benefit brought by hot-deformation processes. Since the optimization of hot-deformation processes is key to obtaining magnets with superior performance, studies on controlling the material flow and process parameters are important. At a time when finite element (FE) simulation is not as powerful as in today’s environment, studies on the material flow could only be carried out through microstructural observations (optical and scanning electron microscopies) and crystal morphology (X-ray diffraction, electron backscatter diffraction pole figure). Traditionally, X-ray diffraction analyses were adopted as proof of HD magnets being well oriented [6,13–17]. By comparing (dividing) the peak intensity values of (0 0 4), (0 0 6) and (1 0 5), one could evaluate the orientation of HD magnets. Intensity values describe the crystal morphology in an analyzed sample, and are hard to correlate with macro-scale material flow or manufacturing process conditions. On the other hand, material flow estimation based on plasticity theories or mechanics models could only obtain results such as strain or stress distributions on deformed bodies. Therefore, more and more studies focused on microstructural observations using advanced analytical instruments, and the observation of macro-scale material flow was ignored. With the improvement of FE simulation modelling and analytical techniques nowadays, it is possible to observe material flow using FE simulation, and correlate with experimental results.

Besides conducting hot-deformation experiments, some of the research works focused on the modelling of hot-deformation processes of Nd-Fe-B magnets. Guruswamy et al. [4] utilized a modified flow stress equation along with the data from Yoshida et al. [3] to describe the deformation behavior of HD Nd-Fe-B magnets. Simulation of HD Nd-Fe-B magnets was conducted to illustrate the strain states of die-upset specimens. They concluded that the maximum principal strains and effective strain showed a maximum value at the central part of the deformed specimen. This result was in agreement with the variation of texture they found in the HD Nd-Fe-B magnets. Years after the first modelling work had been accomplished, Wang et al. [5] performed a 3D finite element simulation of the hot-deformation process of Nd-Fe-B magnets. Yoshida’s data were adopted in that research for evaluating the stress and strain states of backward extruded ring magnets. They also tried to correlate the magnetic properties of ring magnets with strain data, and found the same phenomenon: high effective strain values give high Br values, which yield to high (BH)_max_ values. Lai et al. [6] from the same research group continued the modelling work and the comparison between simulation and experimental results. They found more direct correlations among anisotropic structure formation, remanence enhancement, and highly effective strain values during the hot deformation of Nd-Fe-B magnets.

When it comes to modelling, it is important to identify the applicability of theories adopted in the model. For example, Wang and Lai’s simulation model was based on macro-scale mechanics (material deformation obeys continuum mechanics). The influence of grain boundary behavior was neglected under this scale. On the contrary, when it comes to simulating the magnetization process of material (micro-scale), the point of interest is the mechanism and material behavior among grains or magnetic domains. Liu et al. [7] studied the effect of grain size on the coercivity of HD Nd-Fe-B magnets, and proposed a micromagnetic simulation model to illustrate the magnetization configuration and domain wall propagation. The model and results could help people to understand what activities occurred among grains during magnetization. However, it is still difficult to correlate the micro-scale results with the macro-scale phenomenon directly.

In this work, a macro-scale model is proposed to illustrate the effect of HD processes on the anisotropic structures in HD Nd-Fe-B magnets. It shows how external load and temperature would influence the strain distribution in HD magnets, and coincides with the magnetic property measurement results.

## 2. Modelling for hot-deformed Nd-Fe-B magnets

Nowadays, FE simulations are widely used in analyzing the behavior of materials during plastic deformation for evaluating different processes. Traditional FE simulations of plastic deformation were based on some assumptions, which are: (1) continuum mechanics: the volume of deformed parts remained the same throughout the deformation process; (2) plasticity theories: through solving a series of equations with the boundary conditions from the real deformation situation, the changes in different physical variables could be computed; and (3) governing equations: for the deformation of materials, flow rule (for example, stress-strain relation or stress-strain rate relation) should be well-defined in order to obtain an accurate result. Also, material behavior under different temperatures or strain rates should be defined if the material exhibited different behavior.

### 2.1. Flow rule selection

The flow rule of HD Nd-Fe-B magnets was separated into two types. Flow stress equations describe the material’s behavior by showing the relations between stress and strain values, and the simplest form of flow stress equation would be:


(1)
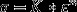



where σ is effective stress, ε is effective strain, K and m are material constants.

In plasticity theories, effective stress and effective strain are defined according to invariants of the stress and strain tensor. When a deformable material underwent complex external loading, yielding and fracture of the material could be predicted by analyzing its stress and strain states. Effective strain is regarded as an index that helps to evaluate the magnitude of plastic deformation of material, and how close it is towards yielding or fracture. The definition of effective strain is:


(2)
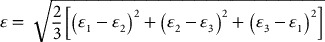



where 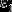, 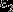, 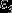 are strain components along three principal directions.

Besides the basic form of flow stress equation, there are many modified forms that tried to introduce parameters such as strain rate and temperature into the equation. The merit of adopting the flow stress equation in simulation is that the material constants could be determined through a standardized experiment procedure.

When it comes to deformation behavior under high temperature, some may introduce the creep model to explain the deformation mechanism of materials. The mechanism could be shown in the form of Arrhenius’ equation, which is:


(3)
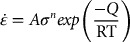



where 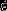 is strain rate, Q is activation energy, T is processing temperature, R is gas constant, A and n are material constants.

Arrhenius’ equation describes the material behavior in the form of strain rate to stress, and the influence of active energy and temperature of material is considered. Although this form gives a comprehensive view on some of the parameters that would affect the material flow, the determination of parameters A, n, and Q through experimental methods are more difficult compared to that in the flow stress equation. Therefore, the flow stress equation is adopted in this article.

### 2.2. Modelling of material flow in finite element simulation

HD Nd–Fe-B magnets are usually made by ultra-fine grain ribbons. The ribbons are hot pressed under elevated temperature (the so-called MQ2 magnet), followed by the HD process around 700–800 °C. Usually, the ribbons come in a well-controlled length-thickness aspect ratio. The thickness of ribbons is usually around 20 μm, with a grain size around 30 nm. After the HD process, the length of ribbon increases, and anisotropic grain growth can be observed.

One point of interest in this work is to illustrate the material flow during the HD process of Nd-Fe-B magnets in the simulation environment. First of all, the flow stress equation was adopted in the model to describe the deformation behavior of HD Nd-Fe-B magnets. The constants in the flow stress equation under high temperature were determined through upsetting experiments [8]. Since the equation is applicable under macro-scale, the simulated results should be compared with the geometries and alignment of the ribbons, not the grains. Second, since the geometry variation of ribbons is small, the specimen before HD is assumed to be isotropic. By introducing plasticity theories, the influences of external load, strain rate, friction condition, and temperature towards the ribbons’ orientation should be illustrated, and shows how they would affect the anisotropic texture in HD Nd-Fe-B magnets.

**Figure 1.**
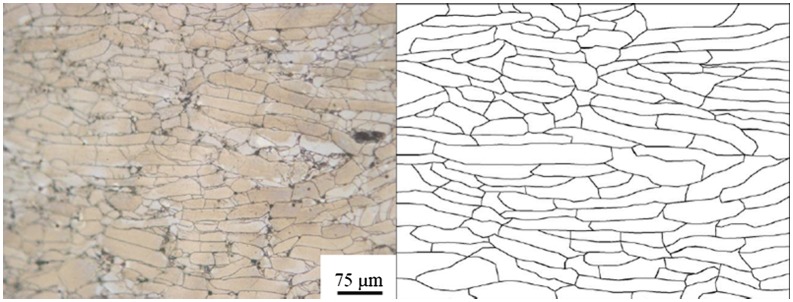
Ribbon pattern established from an OM image.

**Figure 2.**
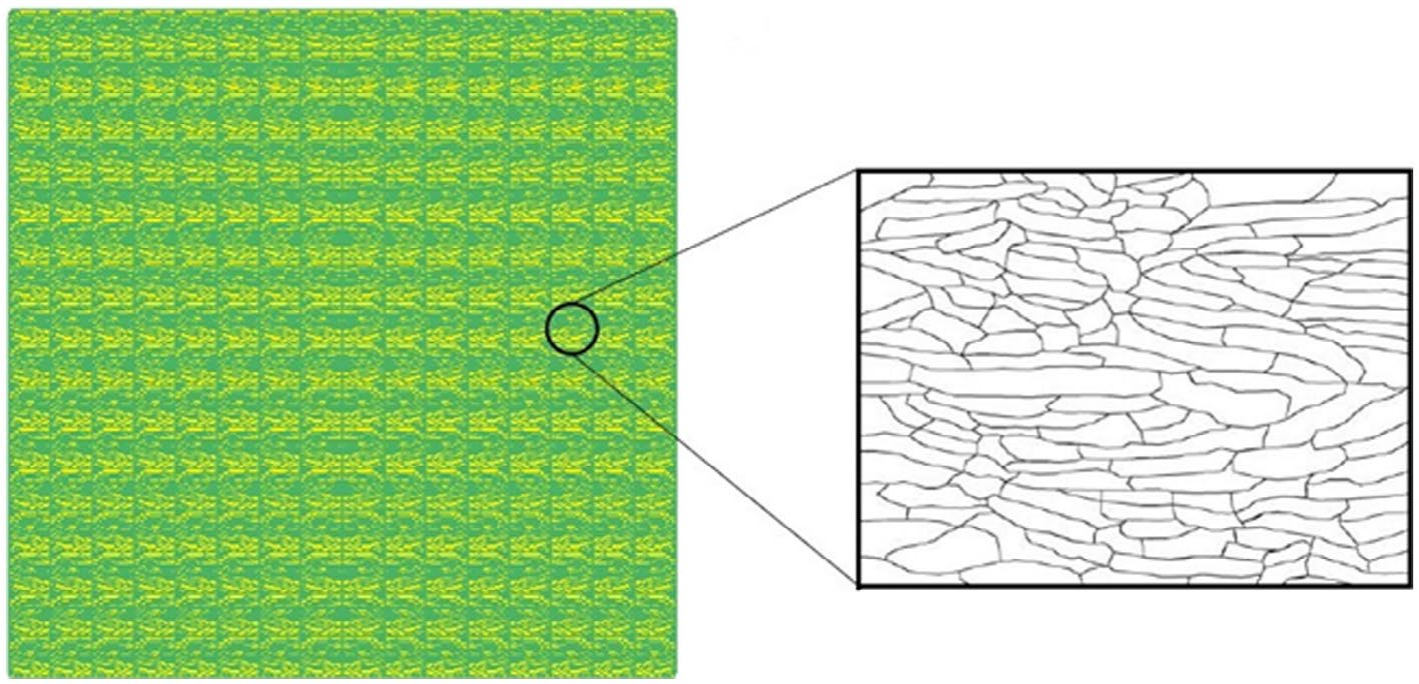
Ribbon pattern deployed in an MQ2 magnet model for HD simulation.

To visualize the material flow of HD Nd-Fe-B magnets, one must establish a ribbon pattern from MQ2 magnets, and calculate their changes after HD processes. Since the MQ2 magnet is regarded as isotropic material, an identical pattern of the compacted ribbons was observed by optical microscope (OM) at a random place in the specimen, as shown in Figure 1. The OM image is a section view taken from the central part of the MQ2 magnet, and then re-plotted in computer-aided design (CAD) software, which became a series of node points with 2D coordinate information (PAT file format). The pattern information could be imported into simulation software, and became the nodal points. Figure 2 illustrates the model of the MQ2 magnet in a simulation where the nodal points had been deployed according to the OM images. Once the simulation was accomplished, the deformed pattern could be analyzed through the post-processing software.

**Figure 3.**
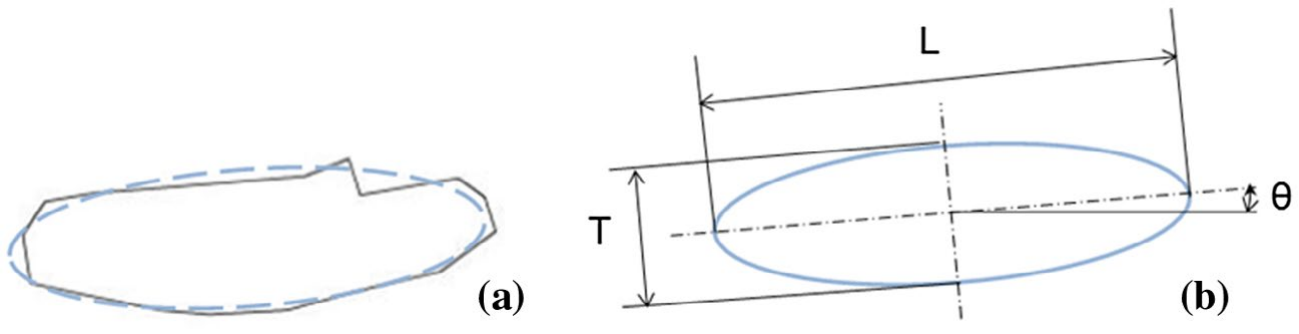
Eclipse fitting for ribbon geometry and tilt angle.

Another important part of modelling is to identify the ribbons before and after the HD process. Unlike powders, the ribbons used for the HD process are usually irregular in the cross-section view. Therefore, the eclipse fitting method is adopted to analyze the dimensions and tilting angle of the ribbons, as shown in Figure 3. In the default coordinate setting, the cross-section of the MQ2 magnets is the X-Z plane, and compressive load would come from the Z-axis. The tilting angle, θ, of the ribbons is defined by the angle with regarding to X-axis. The contour of the ribbons is described by the length of the major axis (L) and length of the minor axis (T).

Table 2 lists the average dimensions measured from MQ2 magnets. From the results, it could be verified that the aspect ratio is about the same at different positions on the cross-section of MQ2 magnets. The ribbons’ average aspect ratio is 0.200, and the standard deviation of the aspect ratio at different positions of the MQ2 magnet is 0.0045. This result supports the assumption of isotropic material in the simulation model.

**Table 2.** Variation of the ribbons’ aspect ratio in MQ2 magnets.

MQ2 Magnet	Major axis, L (μm)	Minor axis, T (μm)	Aspect Ratio (T/L)
Top & Bottom	97.96	19.91	0.204
Middle	95.32	19.42	0.203
Left & Right	98.18	19.02	0.194
Corners	93.22	18.77	0.201
Average	96.17	19.28	0.200
Standard Deviation	0.0195	0.4981	0.0045

In this work, the digital patterns of the ribbons were established by AutoCAD 2014. Pattern files could be imported into packaged simulation software DEFORM-3D v10. The models of two flow rules mentioned in the previous section were built in the software, and users could adjust the material constants freely. In this work, Yoshida’s data [3] were adopted for the input of constants in Arrhenius’ equation. Comparisons with models using the flow stress equation were made to illustrate their differences.

After the simulations were accomplished, the ribbon patterns of HD magnets could be shown in the post-processing program. The macro-scale material flow could be compared with OM images. Also, nodal data with deformation information were exported for further analyses of the ribbons’ average aspect ratio and tilt angles. The eclipse fitting of ribbons was accomplished by an in-house developed program compiled by MATLAB software. The program could compute statistics for the ribbon aspect ratio and tilting angle. This information should be helpful for evaluating die designs and determining process parameters.

## 3. Results and discussion

### 3.1. Influence of effective strain toward material flow and material properties

**Figure 4.**
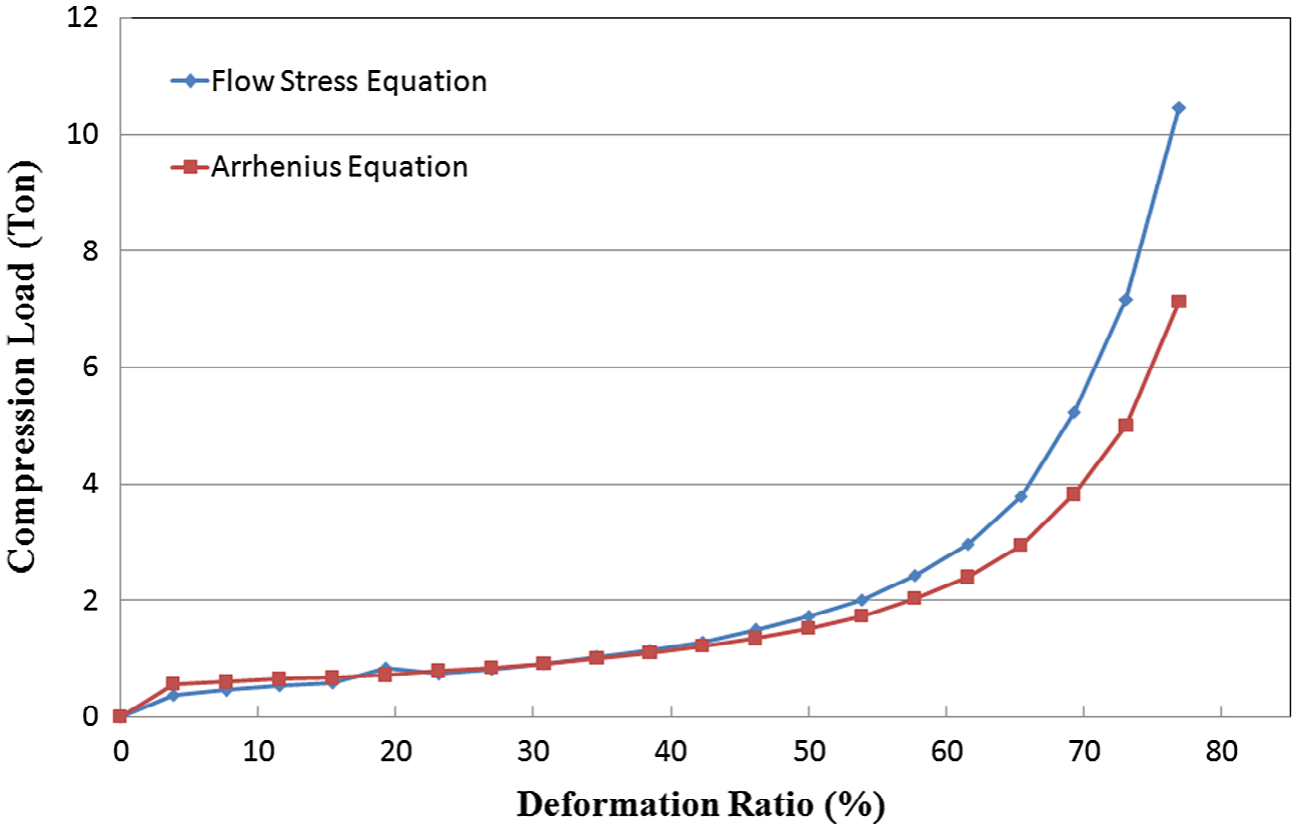
Comparison of load data from two models.

**Figure 5.**
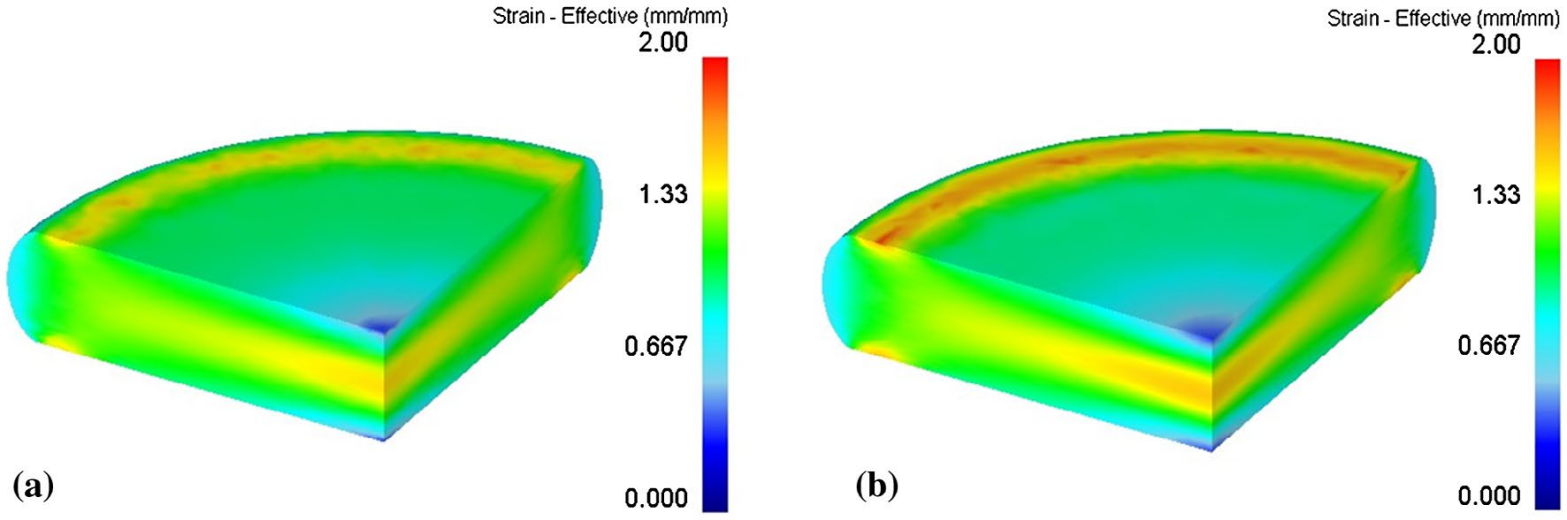
Distribution of effective strain on HD magnets with a 70% deformation ratio, using (a) flow stress equation and (b) Arrhenius’ equation.

Simulations of HD process under the same conditions (MQ2 billet diameter 15 mm, height 13 mm, processing temperature 750 °C, deformation velocity 5x10^−1^ mm/s) were carried out using the above-mentioned two flow rules. A load to deformation ratio diagram could be plotted according to simulation results, as shown in Figure 4. When the deformation ratio is less than 40%, the simulated load data were almost the same. However, when the deformation ratio exceeds 50%, the load predicted by Arrhenius’ equation became less than the flow stress equation. However, judging from the stress-strain curves in previous research, we do not see an obvious softening effect at the high strain region. If simulation results are used for evaluating the required load in the HD process, one needs to be careful that the Arrhenius equation model may deliver an underestimated load result. However, the distribution of effective strain states from the two rules was quite the same, as shown in Figure 5. Both of the models showed the same distribution of effective strain values, and coincide with Yoshida’s results [2]. The flow stress model predicts the effective strain at the central part of the HD magnet is around 1.50, while the Arrhenius equation model gives a result of 1.56.

The relations between strain values and magnetic properties were first discussed by Yoshida et al. [2]. From their results, Br values of HD magnets increased from 8kG to 12.8kG, as the compressive strain values increased from 0 to above 1.5. The results indicated that compressive strain contributes to anisotropic grain growth (along an easy-magnetization axis). A linear increase of Br values towards strain values could be observed when the strain value increases from zero to 1.0. When the strain values increased from 1.0 to 1.5, an increase in Br values could still be observed, but in a much gentler slope. Lai et al. [6] also reported an increase in Br values from 13.2kG to 14.2kG when the strain values vary from 0.95 to 1.70 for HD magnets. For backward extruded ring magnets, Grunberger et al. [10] illustrated the dependence on the axial position of a ring sample towards measured Br values. Br values measured from the middle region of the ring magnet varied from 12.5kG to 12.9kG along the axial direction. Wang et al. [5] further discussed the dependence of effective strain towards Br values using FE simulations. In their observation, the Br value reached above 11kG at the point with a strain value of 1.35. At points with strain values around 1.8 to 1.9, the Br values are all above 12kG. From these results, it could be concluded that a strain value above 1.0 is preferable if high Br values are reached in hot-deformed Nd-Fe-B magnets.

### 3.2. Material flow during hot-deformation of Nd-Fe-B magnets

FE methods can model the 3D distribution of strain states after HD processes. However, it is hard to directly correlate with the magnetic property measurement results. The main problem when calculating strain states in theories is the values represent the deformation degree of a material within a small area (unit area or infinite small area). However, magnetic property measured from specimens is an average value contributed by each and every material within this area.

**Figure 6.**
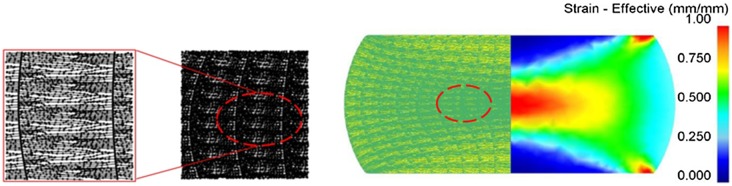
Simulation results of material flow under a 40% deformation ratio.

In this work, the connection between strain states and preferred orientation of ribbons in HD magnets is shown through the ribbons’ aspect ratio and tilting angle. It is proved that Nd-Fe-B magnets could exhibit plastic deformation capability under high temperature and slow strain rates. If grain sliding and rotation governs its deformation behavior, external load may play an important role during the formation of preferred orientation. Under the influence of external load, ribbons in MQ2 magnets might also change their geometry and orientation. Figure 6 shows the effective strain distribution when the deformation ratio of the MQ2 magnet reached 40%. Effective strain at the central part of the magnet reached 1.00, which would give a good magnetic property. At the top and bottom of the HD magnet, the material flow is hindered due to the friction between specimen and die surface, so the strain values are low. The variation of aspect ratios (T/L) in different positions is shown in Table 3. Before deformation, the average aspect ratio is around 0.2. Under 40% of height reduction, the simulated aspect ratio becomes 0.081, which represents thin ribbons where their length is about 12 times their thickness. This result also coincides with the commonly observed phenomenon that material at the central part of HD magnets would have the best alignment and magnetic property [6,16].

**Table 3.** Variation of aspect ratio before and after hot deformation.

	MQ2 Aspect ratio	HD magnet Aspect ratio	Difference
Top & Bottom	0.204	0.123	–40.7%
Middle	0.203	0.081	–60.9%
Left & Right	0.194	0.120	–38.1%
Corners	0.201	0.116	–42.2%
Average	0.200	0.110	–45.1%

**Figure 7.**
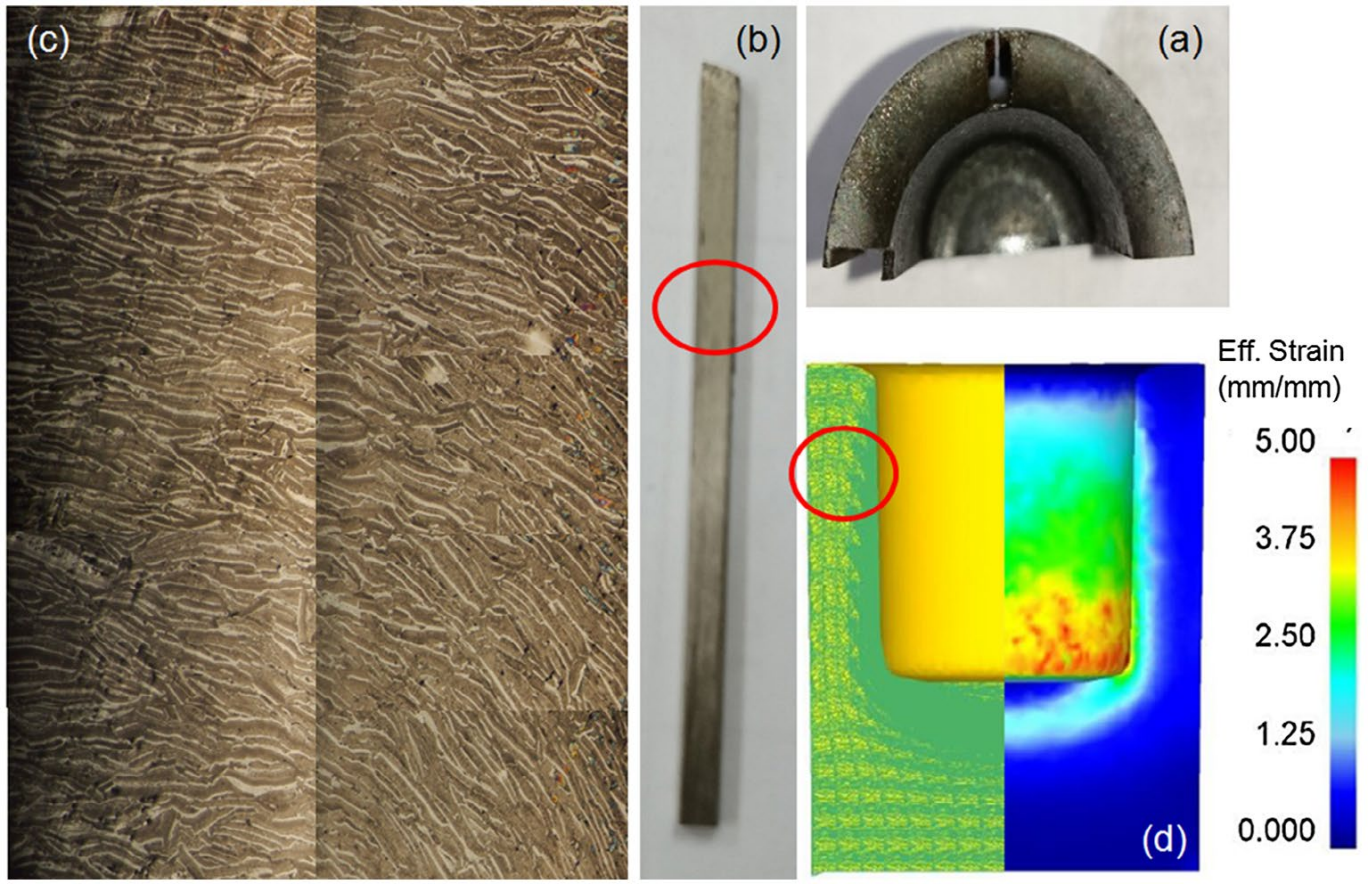
Material flow of backward extruded ring magnets: (a) hot-deformed Nd-Fe-B ring magnets; (b) sample for OM observation; (c) material flow in red circled area; (d) simulative strain distribution and ribbon orientation.

The material flow in cylinder upsetting is simple and symmetric, so the prediction of ribbon orientation may be easy. When it comes to a backward extruded ring, punch geometry would have more influence on the material flow of the deformed specimen. Figure 7 shows the experimental and simulative material flow of a backward extruded ring magnet. First, a Nd-Fe-B ring magnet was obtained by a backward extruded HD process, as shown in Figure 7(a). A cross-section view of the ring magnet was observed, and a sample taken along the height direction (shown in Figure 7(b)). Figure 7(c) shows the OM image of the extruded material. Simulation results such as effective strain distribution and the material flow of corresponding conditions were acquired for comparison, as shown in Figure 7(d).

From Figure 7(c), it could be seen that the orientations of ribbons on the right-hand side (contact with the punch) and left-hand side (contact with the mold) are very different. Material contacting the punch would have larger effective strain due to compressive stress and friction force. On the contrary, the contact stress and friction on the outer position is less, which leads to lower strain values and worse oriented structure. The orientations of the ribbons could also be influenced by the geometry of the punch. Simulated material flow showed the same tendency as the OM observations shown in Figure 7(d). In this case, the draft angle of the punch in this die set is designed to be 2°. Different draft angle and punch head geometry would introduce different stress states, which would lead to a different orientation of the ribbons.

**Figure 8.**
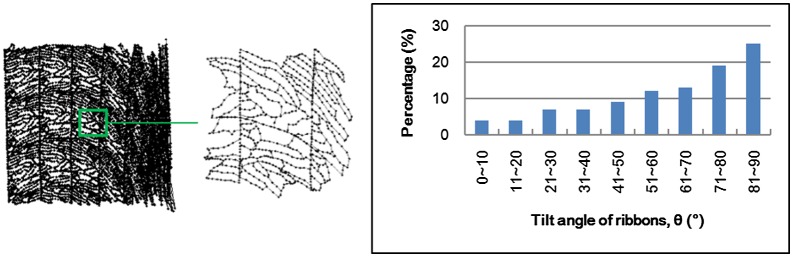
Simulated material flow and distribution of ribbons’ tilt angle.

**Figure 9.**
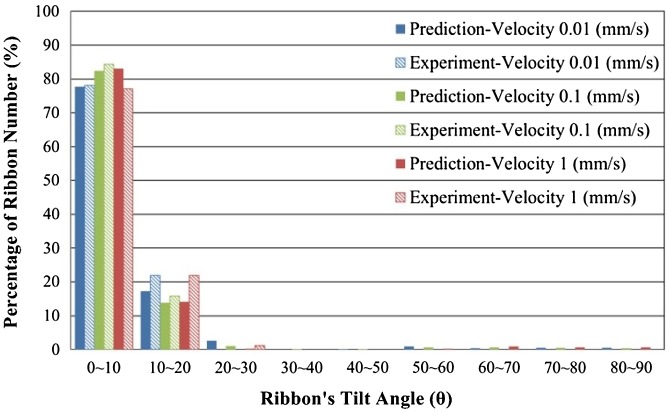
Comparison of experimental and simulated ribbons’ tilt angle distributions at top position of cylinder upsetting magnets.

**Figure 10.**
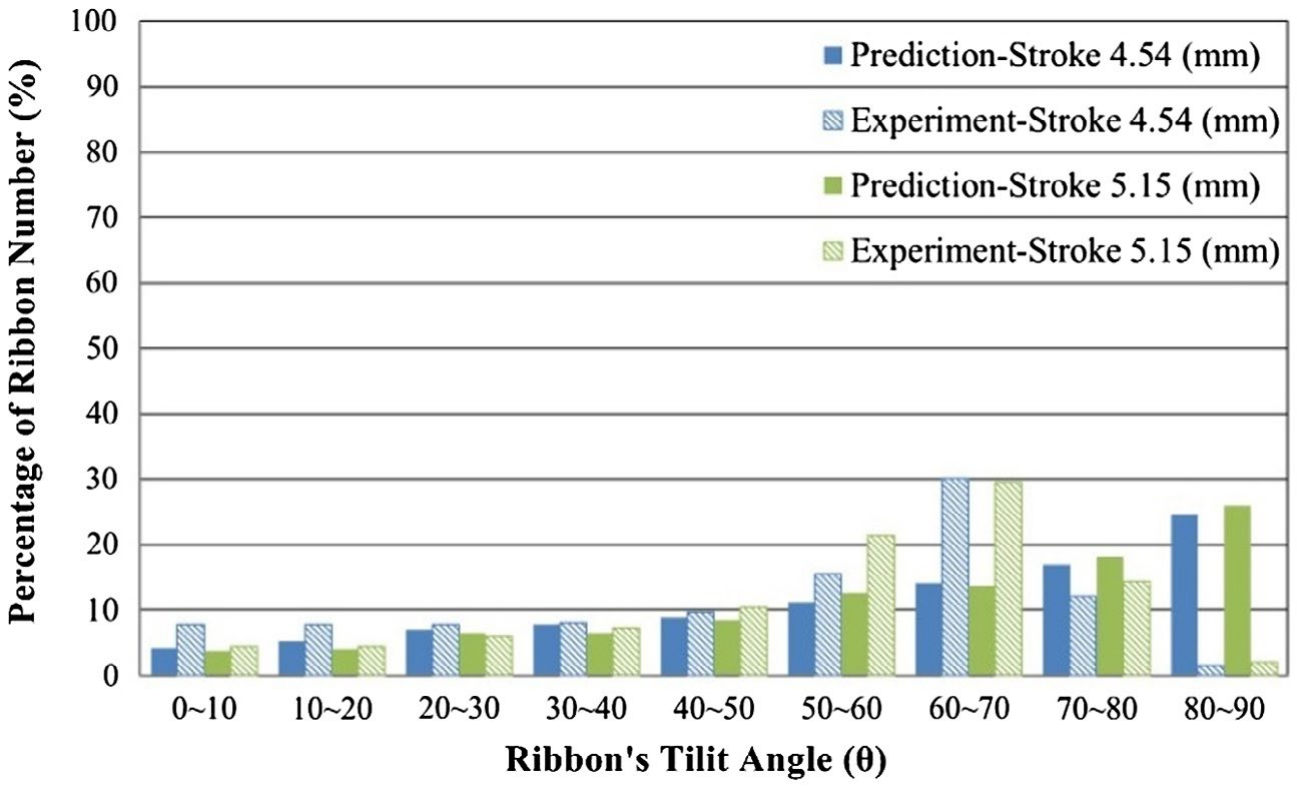
Comparison of experimental and simulated ribbons’ tilt angle distributions at the middle of the ring area in backward extruded ring magnets.

Figure 8 shows the simulated material flow and the statistics of the ribbons’ tilt angles. For HD ring magnets, the direction of material deformation is the same with the punch. An easy magnetization axis of the ring magnet should be aligned with the radial direction. Judging from the statistics shown in Figure 8, about 25% of the ribbons are aligned with the desired direction (>80°). Nearly 30% of the ribbons are not well-aligned as the tilt angles do not exceed 40°. Figure 9 and Figure 10 show the statistics of the ribbons’ tilt angle computed from experimental observation (OM images) and simulated results. Figure 9 shows the orientation of the material at the top of the HD magnets. Upsetting tests with three different punch velocities were performed to test the influence of strain rate towards material flow. From both experimental and simulated results, most of the ribbons were aligned to the Y-axis due to the horizontal material flow behavior. Table 4 shows the error percentage of the average aspect ratios obtained from experiments and simulated results. The error percentage of the average aspect ratio in the top area of HD magnets ranges from -6.1% to +13.4%, indicating that at a slower deformation speed, the influence of grain growth and grain boundary mechanics becomes more significant. Models based on macro-scale mechanics may be too simplified to predict material flow under this scale.

**Table 4.** Comparison of experimentally observed and simulated ribbons’ aspect ratio in HD magnets.

Punch Speed	OM observed Aspect ratio	Simulated Aspect ratio	Error percentage (%)
1.00 (m/s)	0.132	0.124	–6.1%
0.10 (m/s)	0.109	0.123	+12.8%
0.01 (m/s)	0.119	0.135	+13.4%

Figure 10 illustrates the distribution of the ribbons’ tilt angle in the middle region of the backward extruded ring magnet. The observation position is the same as the red circled area shown in Figure 7(b). Since external loading exerted on the material in this area is along the X-axis (horizontal direction), ideal material flow should be aligned to Y-axis, which gives a tilt angle around 90°. However, we could see the distribution of the ribbons’ tilt angle ranges from 0° to 80°, indicating that the orientation is not as good as that in HD magnets. Similar observations could also be found in a previous work [10]. In backward extrusion processes, the friction effect between two contact surfaces is more severe compared to the upsetting process. Also, the friction force induced by the punch is much higher than that from the dies, which results in the uneven strain distribution on the deformed ring parts. When the tilt angle exceeds 70°, the ribbons in OM images are severely elongated and can hardly be recognized by the image processing technique. This is the main reason that the percentage of the ribbons’ number is low at the range of 80° to 90°. However, the results correspond when the ribbons’ tilt angle is less than 50°.

With this material flow evaluation model, engineers could predict if the die designs are ideal for creating sufficient preferred orientation of the ribbons and the grains before spending time conducting experiments. Furthermore, this material flow model could also be utilized if engineers decide to cut off the material with poor magnetic property.

## 4. Conclusions

In this work, a methodology for simulating the material flow of hot-deformed Nd-Fe-B magnets is proposed. By using the methodology, the distribution of ribbon aspect ratio and their tilt angles was quantified and analyzed both in OM observation and simulations. This methodology will help researchers understand the mechanism of texture formation and the evolution of magnetic properties. Based on macro-scale plastic deformation theories, the simulated material flow is correlated with experimental observations. Material flow during upsetting and backward extrusion of Nd-Fe-B magnets was analyzed. The ribbons’ aspect ratio and tilt angle were utilized as an index representing the material flow of HD magnets. For the upsetting process, the aspect ratio at the central part could be altered from 0.203 to 0.081. Most of the ribbons were aligned to the Y-axis due to the horizontal material flow behavior, with their tilt angles ranging from 0° to 20°. The error percentage of the simulated ribbons’ aspect ratio in the top area of HD magnets ranges from -6.1% to +13.4%, showing good correlation with experimental observations.

## Disclosure statement

No potential conflict of interest was reported by the authors.

## References

[1] Lee RW Hot-pressed neodymium-iron-boron magnets. Appl Phys Lett. 1985;46(8):790–791. 10.1063/1.95884

[2] Yoshida Y, Kinami T, Yoshikawa N, et al Magnetic properties and plastic strains of hot formed Nd-Fe-B magnets. Denki-Seiko. 1990;61(3):193–200. 10.4262/denkiseiko.61.193

[3] Yoshida Y, Kasai Y, Watanabe T, et al Hot workability of melt-spun Nd-Fe-Co-B magnets. J Appl Phys. 1991;69(8):5841–5843. 10.1063/1.347844

[4] Guruswamy S, Wang YR, Panchanathan V Plastic deformation modeling of die-upset process for magnequench NdFeB magnets. J Appl Phys. 1998;83(11):6393–6395. 10.1063/1.367535

[5] Wang HJ, Lin M, Lai B, et al Plastic deformation modeling of backward extrusion process for NdFeB ring magnets. J Magn Magn Mater. 2012;324:1791–1794. 10.1016/j.jmmm.2011.12.040

[6] Lai B, Wang HJ, Zhu MG, et al Simulation of the die-upsetting process of hot-deformed magnets. J Korean Phys Soc. 2013;63(3):320–324. 10.3938/jkps.63.320

[7] Liu J, Sepehri-Amin H, Ohkubo T, et al Grain size dependence of coercivity of hot-deformed Nd–Fe–B anisotropic magnets. Acta Mater. 2015;82:336–343. 10.1016/j.actamat.2014.09.021

[8] Chen YJ, Chang CC, Hsiao PJ, et al Investigation on the Improvement of Magnetic Properties by Hot Deformation Processes for NdFeB Magnets. Key Eng Mater. 2015;626:317–322.

[9] Kasai Y, Watanabe T, Shibata S, et al MQ2 and MQ3 magnets - Improvements in production technology and properties. Denki-Seiko. 1991;62(4):241–251. 10.4262/denkiseiko.62.241

[10] Grunberger W, Hinz D, Kirchner A, et al Hot deformation of nanocrystalline Nd-Fe-B alloys. J Alloys Compd. 1997;257:293–301. 10.1016/S0925-8388(97)00026-1

[11] Kim HT, Kim YB, Kim HS Application of the current-applied pressure-assisted method for anisotropic NdFeB magnets. J Magn. 2000;5(4):130–134.

[12] Kirchner A, Hinz D, Panchanathan V, et al Improved hot workability and magnetic properties in NdFeCoGaB hot deformed magnets. IEEE Trans Magn. 2000;36(5):3288–3290. 10.1109/20.908772

[13] Hioki K, Takano T, Yamamoto T Influence of process conditions on the magnetic properties for hot deformed magnets. Denki-Seiko. 2008;79(2):119–125. 10.4262/denkiseiko.79.119

[14] Hou YH, Huang YL, Liu ZW, et al Hot deformed anisotropic nanocrystalline NdFeB based magnets prepared from spark plasma sintered melt spun powders. Mater Sci Eng B. 2013;178:990–997. 10.1016/j.mseb.2013.06.009

[15] Zhang TQ, Chen FG, Zheng Y, et al Hot-deformed Nd-Fe-B magnets fabricated by dynamic loading with a high maximum energy product. Intermetallics. 2016;73:67–71. 10.1016/j.intermet.2016.04.001

[16] Lee YI, Huang GY, Chang ST, et al. Optimization of the magnetic properties of hot deformed Nd-Fe-B magnets. IEEE Magn Lett. 2017;8:5500204 (4 p.)

[17] Zhu MG, Li W Texture formation mechanism and constitutive equation for anisotropic thermorheological rare-earth permanent magnets. AIP ADV. 2017;7:056236 (6 p.) 10.1063/1.4978700

